# Supplementary material for: Interactions between Malassezia and New Therapeutic Agents in Atopic Dermatitis Affecting Skin Barrier and Inflammation in Recombinant Human Epidermis Model
Source: Int J Mol Sci. 2023 Mar 24;24(7):6171. doi: 10.3390/ijms24076171 (PMC10094540; doi:10.3390/ijms24076171)
Supplement: Supplementary file 1 [file ijms-24-06171-s001.zip › ijms-2291395-supplementary.pdf]

Supplementary Material & Data

Interactions between *Malassezia* and new therapeutic agents in Atopic Dermatitis affecting skin barrier and inflammation in Recombinant Human Epidermis model

1.1. Primer sequence

Species	Primer name	Forward (5'-3')	Reverse (5'-3')
Human	IL-1α	CAG TTC TGC TGA CTG GGT GA	AGG TGC TGA CCT AGG CTT GA
	IL-4	CCT CAC AGA GCA GAA GAC TC	CTC ATG GTG GCT GTA GAA CT
	IL-4Rα	AGG TGG GGT CAT AGC AAC AG	GCA AGC ACA CCT CAT CTC AA
	IL-17	ACC AAT CCC AAA AGG TCC TC	GGG GAC AGA GTT CAT GTG GT
	IL-22	ACA GGT TCT CCT TCC CCA GT	GGT GAT ATA GGG CTG CTG GA
	CCL17	TGT GGT CCA GCA GAG AGA TG	AGG GTG TCC TCT TGG TTC CT
	CCL20	GCT GCT TTG ATG TCA GTG CT	GAT GTC ACA GCC TTC ATT GG
	CCL22	GAA CCT GTG GAA TTG GAG GA	CTG GAT GAC ACT GAG CTG GA
	TSLP	CTC TGG AGC ATC AGG GAG AC	AGG GAA CAT ACG TGG ACA CC
	VEGF	TGC CCG CTG CTG TCT AAT	TCT CCG CTC TGA GCA AGG
	TNF-α	CAC CAC TTC GAA ACC TGG GA	AGG AAG GCC TAA GGT CCA CT
	IFN-γ	GCA GCC AAC CTA AGC AAG AT	GGG TCA CCT GAC ACA TTC AA
	CERS3	AGG ACC ACA CCA GGA GAC AC	AGT GCA AAG TGG GTT GGT TC
	ELOVL1	ACT TGG GAG AGG AGC ACT CA	GAG TAA GCA GCC TCC ACA GG
	GAPDH	GAA GGT GAA GGT CGG AGT CAA	GCT CCT GGA AGA TGG TGA TG

Table S1. Primer sequence

All primers (Table S1) were designed using the coding sequences available on the GenBank database (http://www.ncbi.nlm.nih.gov/Genbank/Genbank Search.html) and were synthesized by Bioneer custom oligo synthesis service (Bioneer, Daejeon, Korea).

2.1. Gene expression of AD marker and cell viability of HaCaT cells.

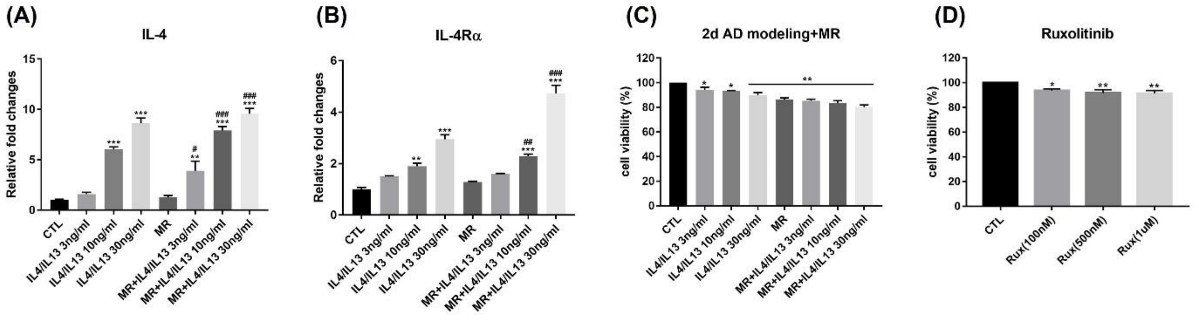

Figure S1. Gene expression of AD marker and cell viability of HaCaT cells. The mRNA expression of (A) IL-4 and (B) IL-4Rα was analyzed by RT-PCR. AD marker genes were upregulated by IL-4/IL-13 in concentrations greater than 10ng/ml. Cell viability of (C) AD modeling with *M.restricta*, and (D) ruxolitinib was evaluated by MTT assay. As the concentration of IL-4/IL-13 increases, cell viability was decreased by 80% in the MR-treated groups. Ruxolitinib had a negligible impact on cell viability. Error bars represent the mean ± SEM, n = 3. Statistically significant at * $p < 0.05$, ** $p < 0.01$, and *** $p < 0.001$ compared to the control (CTL) and # $p < 0.05$, ## $p < 0.01$, and ### $p < 0.001$ compared to the MR-only treated group (MR).

2.2. Analysis of epidermal skin barrier protein and antimicrobial peptides of MR-treated and MR-untreated groups.

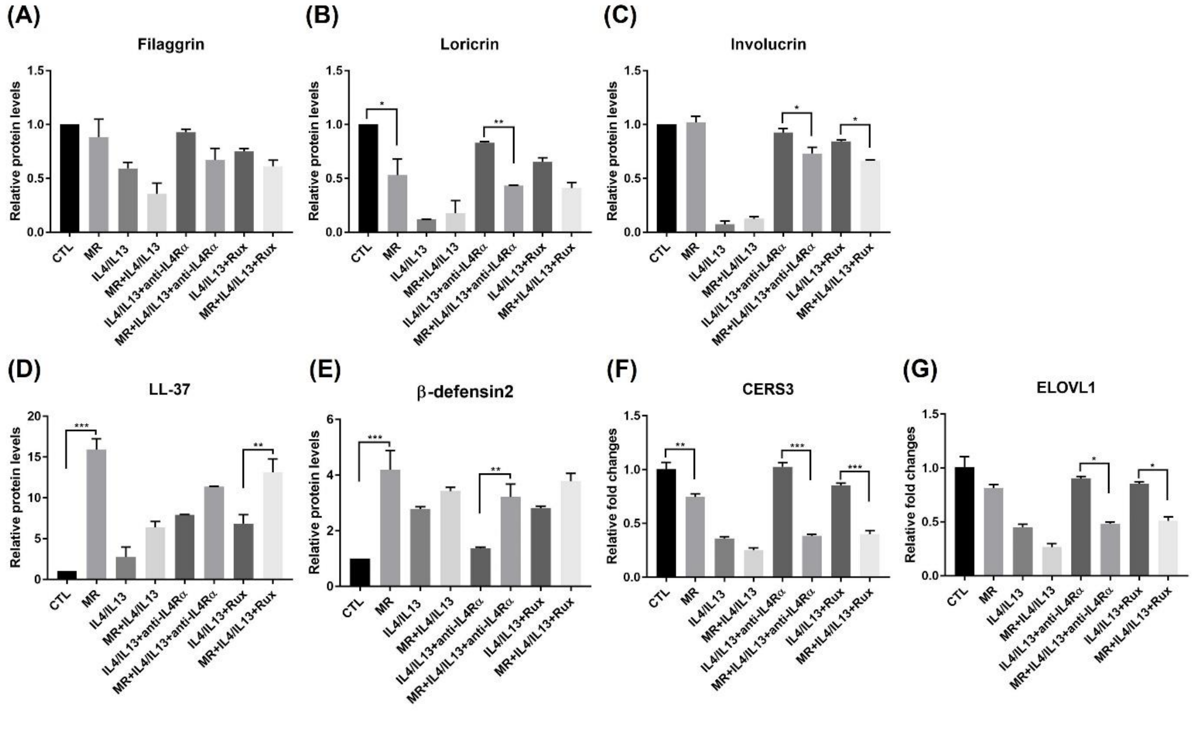

Figure S2. Analysis of epidermal skin barrier protein and antimicrobial peptides of MR-treated and MR-untreated groups. The protein expression of (A) FLG, (B) LOR, (C) IVL, (D) LL-37, and (E) β-defensin2 in RHE was measured by western blotting. The mRNA expression of (F) CerS3 and (G)

ELOVL1 in RHE was measured by PCR. Error bars represent the mean ± SEM, n = 3. Statistically significant at * $p < 0.05$, ** $p < 0.01$, and *** $p < 0.001$ compared to each group.

2.3. TEM image of control group.

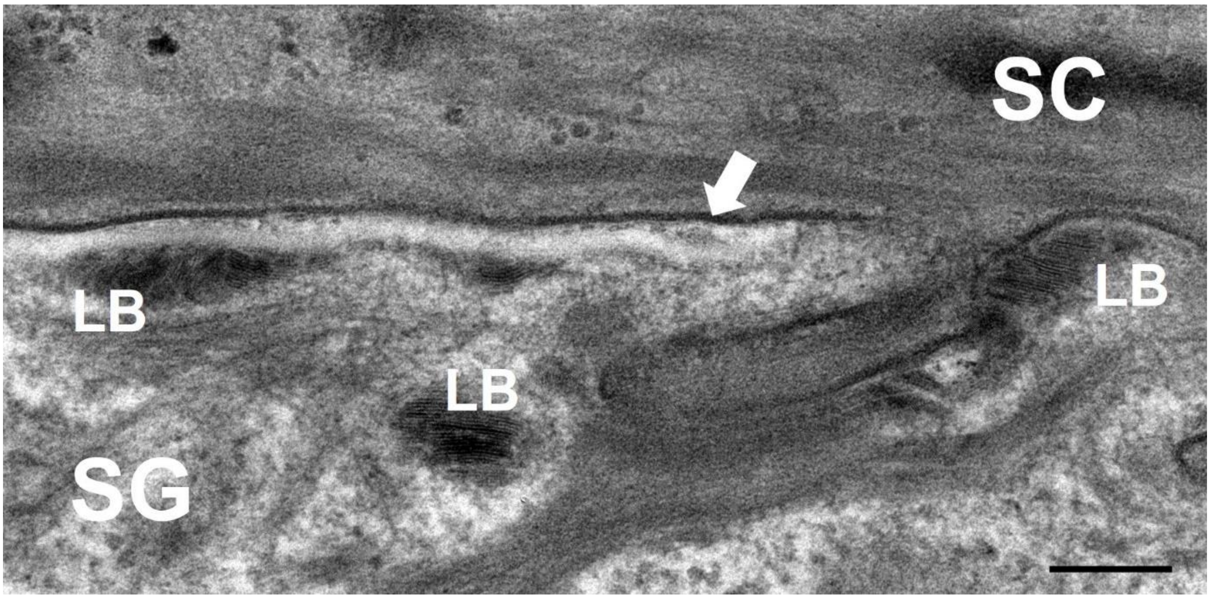

Figure S3. TEM image of control group.

The intercellular lipid layer is filled with lipid lamellae, underneath which is located the lamellae body. SC: stractum corneum, SG: stractum granulm, LB: lamellae body, white arrow: intercellular lipid layer (ILL), Scale bar = 0.2 μm

2.4. Analysis of Th1, Th2, and Th17-related gene expression comparison between MR-treated and MR-untreated groups.

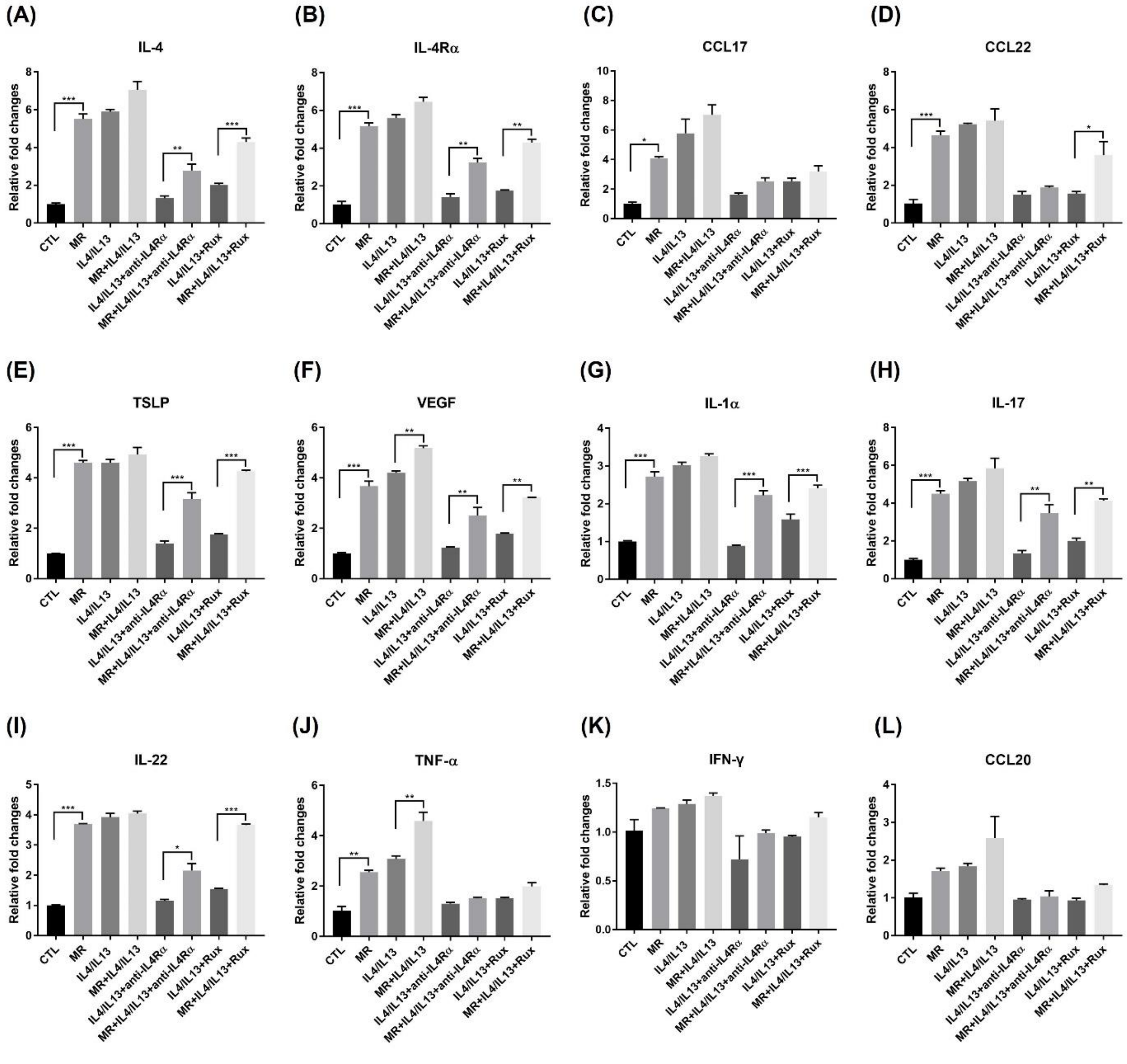

Figure S4. Analysis of Th1, Th2, and Th17-related gene expression comparison between MR-treated and MR-untreated groups. The mRNA expression of (A) IL-4, (B) IL-4Rα, (C) CCL17, (D)CCL22, (E) TSLP (F) VEGF, (G) IL-1α, (H) IL-17, (I) IL-22, (J) TNF-α, (K) IFN-γ (L) CCL20 in RHE by RT-PCR. Error bars represent the mean ± SEM, n = 3. Statistically significant at * $p < 0.05$, ** $p < 0.01$, and *** $p < 0.001$ compared to each group.

2.5. Analysis of Th2, Th17 related proteins comparison between MR-treated and MR-untreated groups.

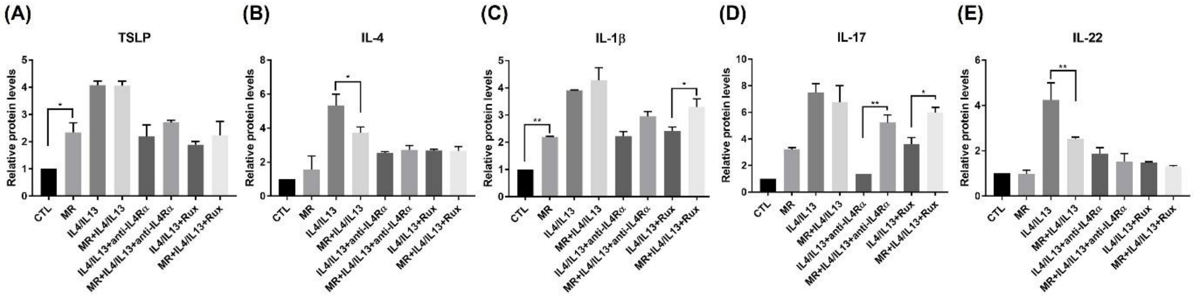

Figure S5. Analysis of Th2, Th17 related proteins comparison between MR-treated and MR-untreated groups. The protein levels of (A) TSLP, (B) IL-4, (C) IL-1β, (D) IL-17, and (E) IL-22 in RHE were measured by western blotting. Error bars represent the mean ± SEM, n = 3. Statistically significant at * $p < 0.05$ and ** $p < 0.01$ compared to each group.

2.6. Analysis of protein levels of JAK/STAT pathway-related molecules comparison in MR-treated and MR-untreated groups.

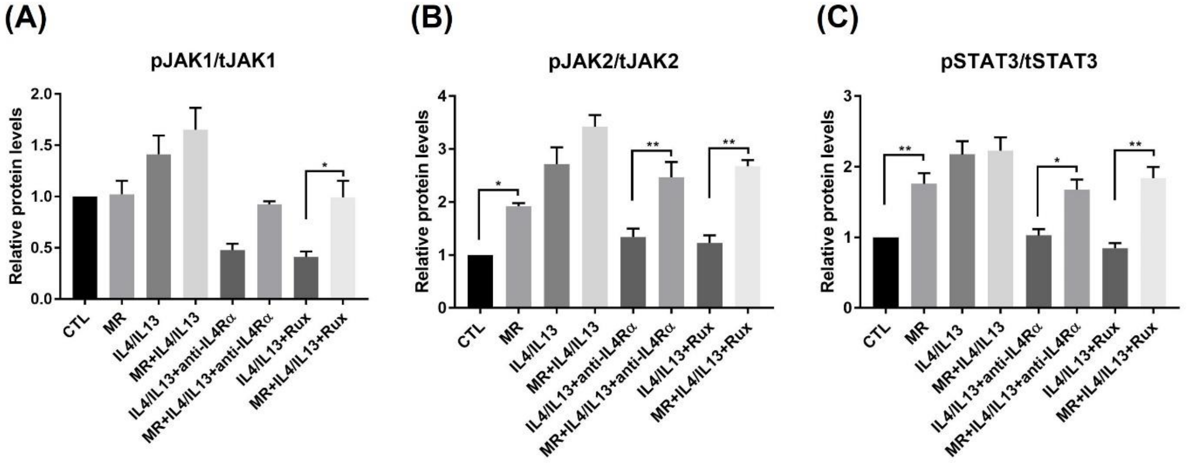

Figure S6. Analysis of protein levels of JAK/STAT pathway-related molecules comparison in MR-treated and MR-untreated groups. The protein expression of (A) pJAK1/tJAK1, (B) pJAK2/tJAK2, and (C) pSTAT3/tSTAT3 in RHE measured by western blotting. Error bars represent the mean ± SEM, n = 3. Statistically significant at * $p < 0.05$, and ** $p < 0.01$ compared to each group.
